# Supplementary material for: The influence of observation sequence features on the performance of the Bayesian hidden Markov model: A Monte Carlo simulation study
Source: PLoS One. 2024 Dec 11;19(12):e0314444. doi: 10.1371/journal.pone.0314444 (PMC11633971; doi:10.1371/journal.pone.0314444)
Supplement: S1 Appendix — (PDF) [file pone.0314444.s001.pdf]

Appendix S1. Starting probabilities of the transition and emission probability matrices.

The starting values of the transition probability matrix were set to:

$$\Gamma = \begin{pmatrix} 0.70 & 0.10 & 0.20 \\ 0.10 & 0.70 & 0.20 \\ 0.10 & 0.20 & 0.70 \end{pmatrix}.$$

The starting values of the emission probability matrices were set to:

	Number of categorical levels		
	Three	Five	Seven
State distinctiveness			
High	$\begin{pmatrix} 0.60 & 0.20 & 0.20 \\ 0.20 & 0.60 & 0.20 \\ 0.20 & 0.20 & 0.60 \end{pmatrix}$	$\begin{pmatrix} 0.60 & 0.10 & 0.10 & 0.10 & 0.10 \\ 0.10 & 0.35 & 0.10 & 0.35 & 0.10 \\ 0.10 & 0.10 & 0.35 & 0.10 & 0.35 \end{pmatrix}$	$\begin{pmatrix} 0.60 & 0.06 & 0.06 & 0.07 & 0.07 & 0.07 & 0.07 \\ 0.04 & 0.40 & 0.04 & 0.40 & 0.04 & 0.04 & 0.04 \\ 0.04 & 0.04 & 0.28 & 0.04 & 0.28 & 0.04 & 0.28 \end{pmatrix}$
Moderate	Excluded	$\begin{pmatrix} 0.60 & 0.10 & 0.10 & 0.10 & 0.10 \\ 0.10 & 0.35 & 0.10 & 0.35 & 0.10 \\ 0.10 & 0.10 & 0.35 & 0.10 & 0.35 \end{pmatrix}$	Excluded
Low	$\begin{pmatrix} 0.60 & 0.20 & 0.20 \\ 0.20 & 0.60 & 0.20 \\ 0.20 & 0.20 & 0.60 \end{pmatrix}$	$\begin{pmatrix} 0.60 & 0.10 & 0.10 & 0.10 & 0.10 \\ 0.10 & 0.36 & 0.09 & 0.36 & 0.09 \\ 0.09 & 0.10 & 0.36 & 0.09 & 0.36 \end{pmatrix}$	$\begin{pmatrix} 0.60 & 0.06 & 0.06 & 0.07 & 0.07 & 0.07 & 0.07 \\ 0.04 & 0.40 & 0.04 & 0.40 & 0.04 & 0.04 & 0.04 \\ 0.04 & 0.04 & 0.28 & 0.04 & 0.28 & 0.04 & 0.28 \end{pmatrix}$
State separation			
High	Excluded	$\begin{pmatrix} 0.56 & 0.11 & 0.11 & 0.11 & 0.11 \\ 0.10 & 0.36 & 0.36 & 0.09 & 0.09 \\ 0.09 & 0.10 & 0.09 & 0.36 & 0.36 \end{pmatrix}$	Excluded
Moderate	Excluded	$\begin{pmatrix} 0.56 & 0.11 & 0.11 & 0.11 & 0.11 \\ 0.10 & 0.36 & 0.36 & 0.09 & 0.09 \\ 0.09 & 0.10 & 0.09 & 0.36 & 0.36 \end{pmatrix}$	Excluded
Low	Excluded	$\begin{pmatrix} 0.56 & 0.11 & 0.11 & 0.11 & 0.11 \\ 0.10 & 0.36 & 0.36 & 0.09 & 0.09 \\ 0.09 & 0.10 & 0.09 & 0.36 & 0.36 \end{pmatrix}$	Excluded

The starting values of the emission probability matrix of the "High" state distinctiveness by "Five" number of categorical levels cell that were re-estimated on the basis of a non-uniform distribution for the third state were finally set to:

$$\begin{pmatrix} 0.60 & 0.10 & 0.10 & 0.10 & 0.10 \\ 0.10 & 0.36 & 0.09 & 0.36 & 0.09 \\ 0.09 & 0.10 & 0.36 & 0.09 & 0.36 \end{pmatrix}.$$
