# Supplementary material for: The influence of observation sequence features on the performance of the Bayesian hidden Markov model: A Monte Carlo simulation study
Source: PLoS One. 2024 Dec 11;19(12):e0314444. doi: 10.1371/journal.pone.0314444 (PMC11633971; doi:10.1371/journal.pone.0314444)
Supplement: S1 Fig — (PDF) [file pone.0314444.s002.pdf]

Fig. S2. Trellis plots of mean absolute bias, mean empirical standard error, and mean bias-corrected coverage of transition and emission probabilities.

Transition probabilities

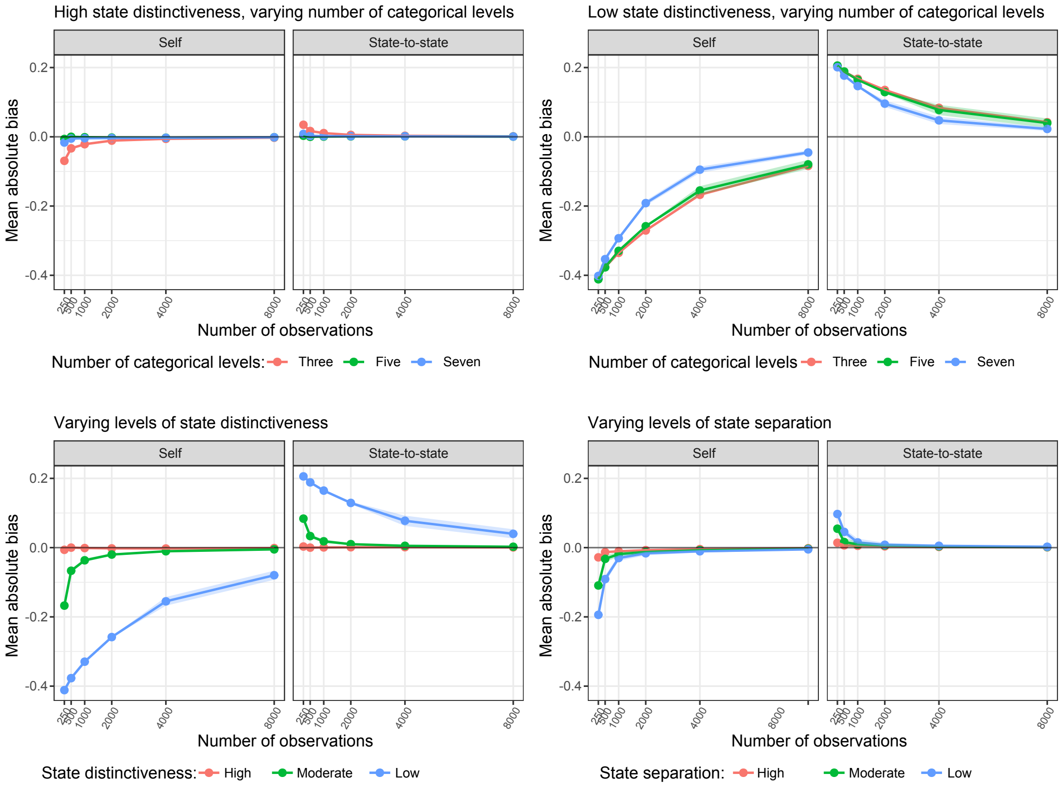

Fig. A. Trellis plot of the mean absolute bias for the estimation of the self and state-to-state transition probabilities. Plot shows the subset of scenarios with high state distinctiveness (upper left panel) and low state distinctiveness (upper right panel) over levels of number of categorical variables (line color) and number of observations, and the subset of scenarios with varying levels of state distinctiveness (bottom left panel; line color) and varying levels of state separation (bottom right panel; line color) over number of observations.

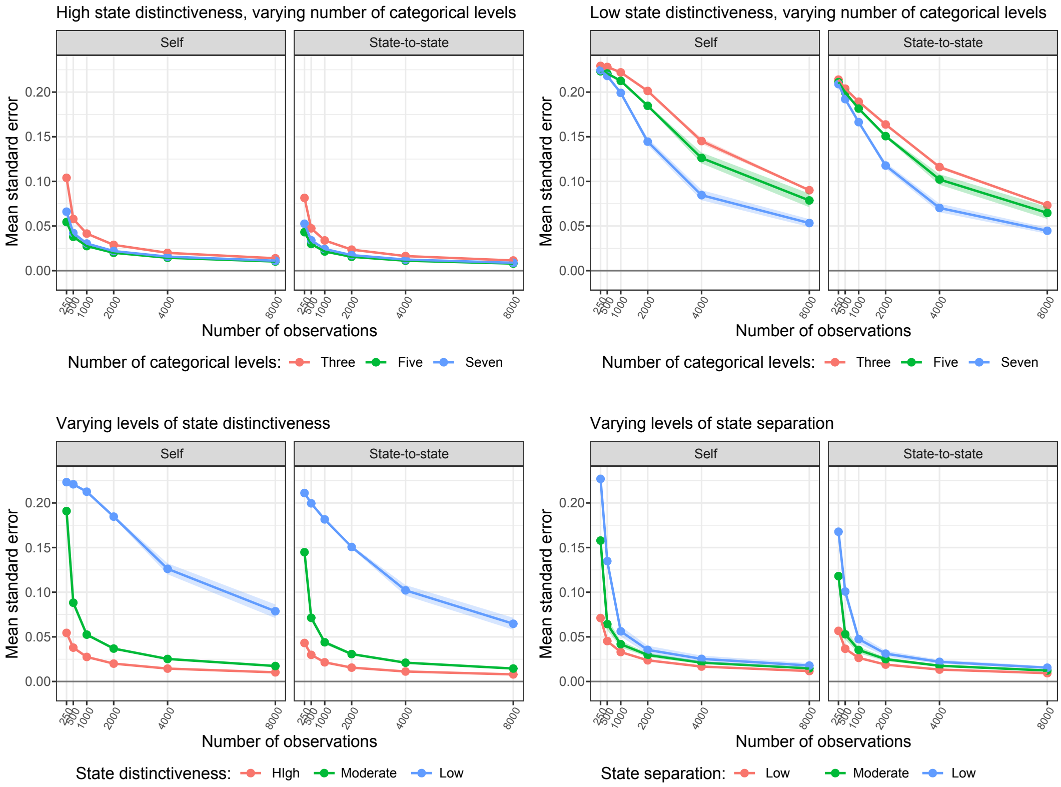

Fig. B. Trellis plot of the mean empirical model standard error for the estimation of the self and state-to-state transition probabilities. Plot shows the subset of scenarios with high state distinctiveness (upper left panel) and low state distinctiveness (upper right panel) over levels of number of categorical variables (line color) and number of observations, and the subset of scenarios with varying levels of state distinctiveness (bottom left panel; line color) and varying levels of state separation (bottom right panel; line color) over number of observations.

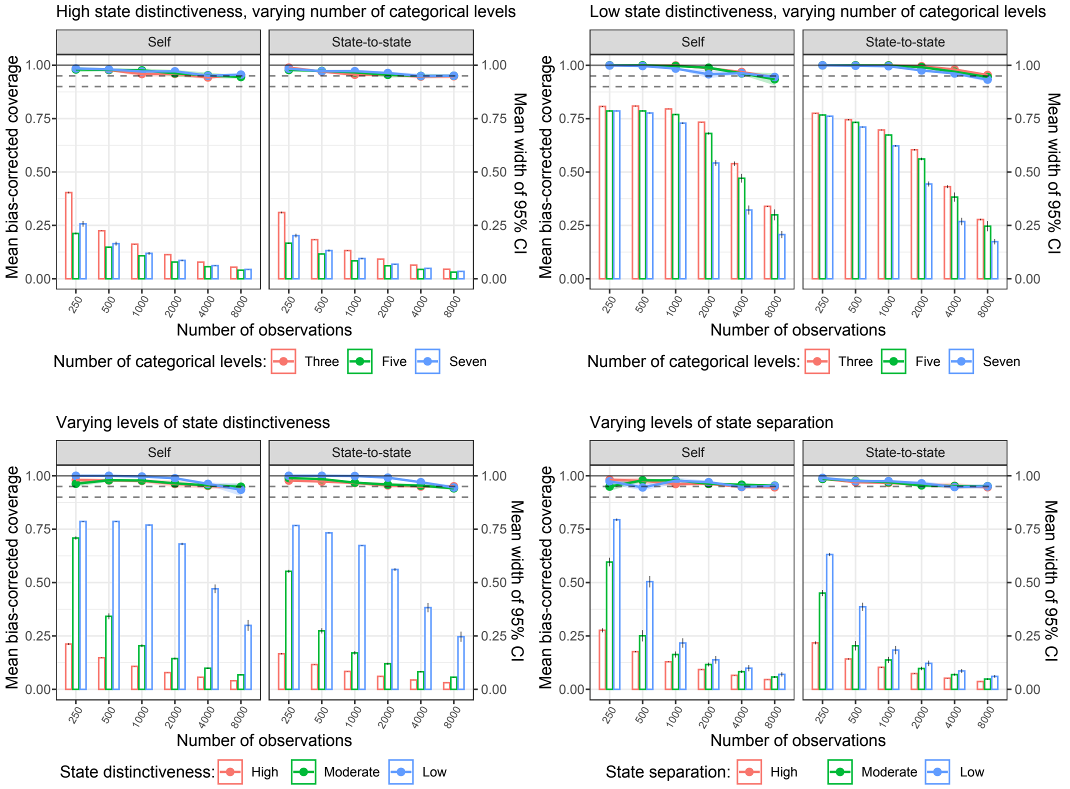

Fig. C. Trellis plot of the mean bias-corrected coverage for the estimation of the self and state-to-state transition probabilities The plot shows the subset of scenarios with high state distinctiveness (upper left panel) and low state distinctiveness (upper right panel) over levels of number of categorical variables (line color) and number of observations, and the subset of scenarios with varying levels of state distinctiveness (bottom left panel; line color) and varying levels of state separation (bottom right panel; line color) over number of observations. Bars represent the mean width of the 95% credibility interval (CrI), right sided y-axis.

Emission probabilities

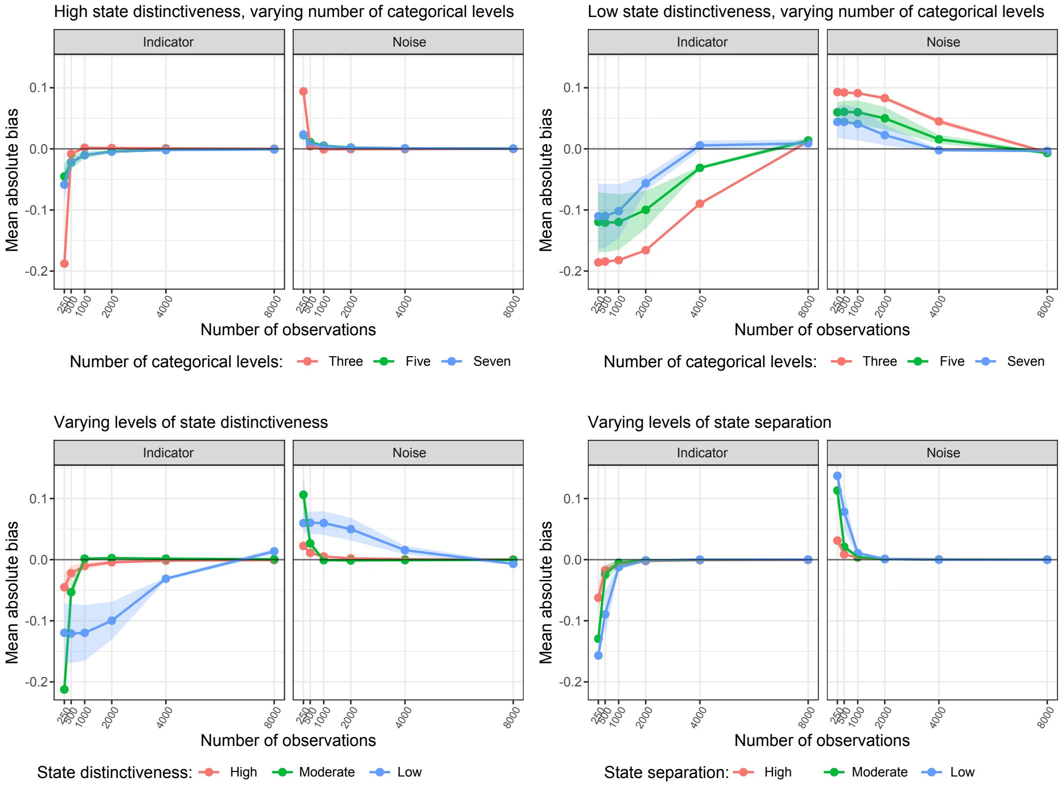

Fig. D. Trellis plot of the mean absolute bias for the estimation of the indicator and noise emission probabilities. Plot shows the subset of scenarios with high state distinctiveness (upper left panel) and low state distinctiveness (upper right panel) over levels of number of categorical variables (line color) and number of observations, and the subset of scenarios with varying levels of state distinctiveness (bottom left panel; line color) and varying levels of state separation (bottom right panel; line color) over number of observations.

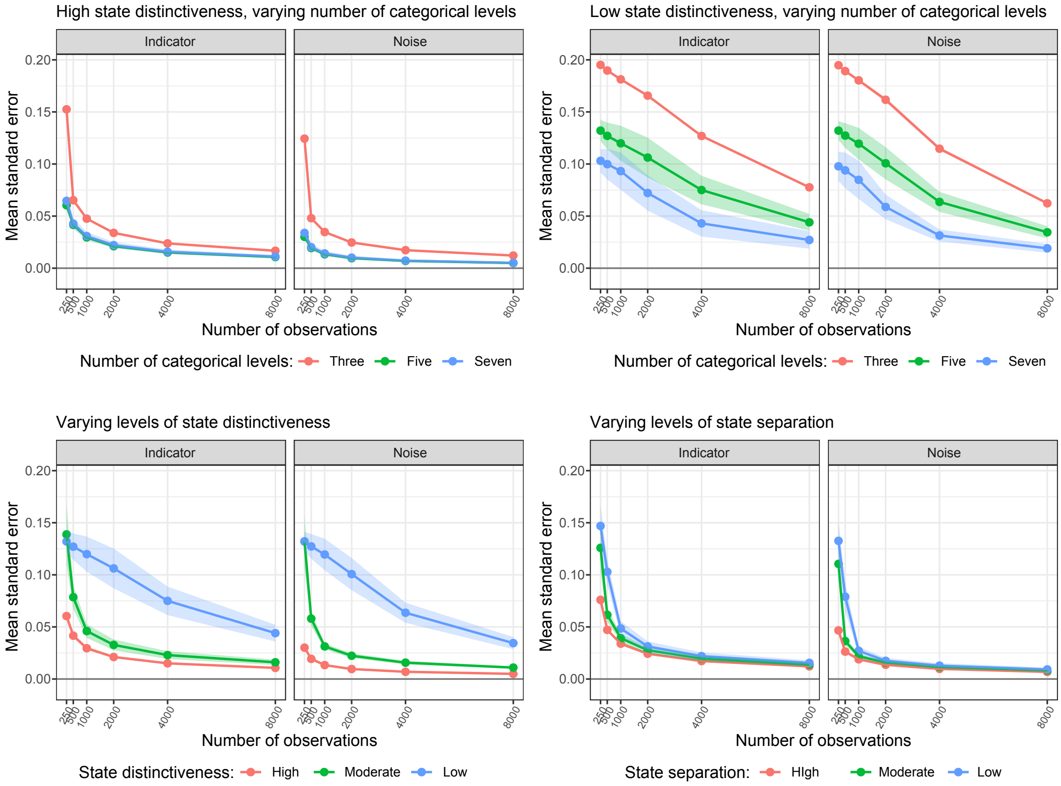

Fig. E. Trellis plot of the mean empirical model standard error for the estimation of the indicator and noise emission probabilities. Plot shows the subset of scenarios with high state distinctiveness (upper left panel) and low state distinctiveness (upper right panel) over levels of number of categorical variables (line color) and number of observations, and the subset of scenarios with varying levels of state distinctiveness (bottom left panel; line color) and varying levels of state separation (bottom right panel; line color) over number of observations.

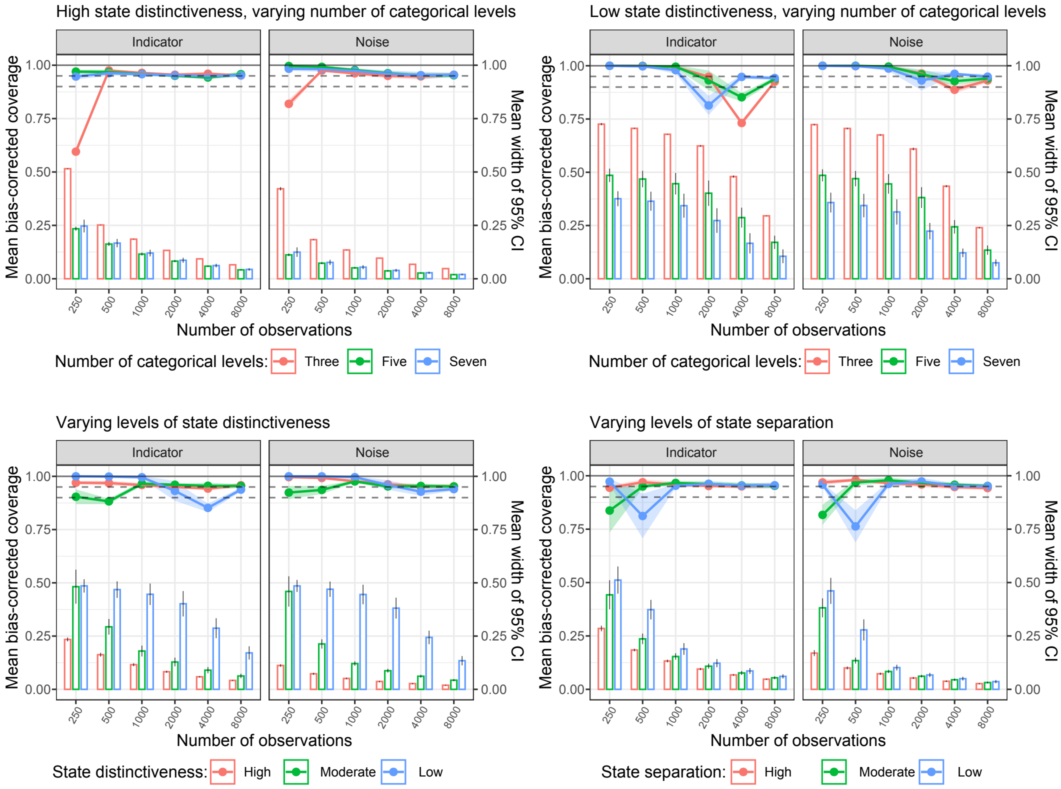

Fig. F. Trellis plot of the mean bias-corrected coverage for the estimation of the indicator and noise emission probabilities. Plot shows the subset of scenarios with high state distinctiveness (upper left panel) and low state distinctiveness (upper right panel) over levels of number of categorical variables (line color) and number of observations, and the subset of scenarios with varying levels of state distinctiveness (bottom left panel; line color) and varying levels of state separation (bottom right panel; line color) over number of observations. Bars represent the mean width of the 95% credibility interval (CrI), right sided y-axis.
